# Efficacy and safety of pharmacological interventions for pruritus in primary biliary cholangitis: A systematic review and meta-analysis

**DOI:** 10.3389/fphar.2022.835991

**Published:** 2022-10-20

**Authors:** Chenyi Xu, Rensong Yue, Xuelian Lv, Shengnan Wang, Mengmeng Du

**Affiliations:** ^1^ Hospital of Chengdu University of Traditional Chinese Medicine, Chengdu, China; ^2^ Xinjin Hospital of Traditional Chinese Medicine, Chengdu, China; ^3^ Qing Dao NO.6 People’s Hospital, Qing Dao, China

**Keywords:** primary biliary cholangitis, pruritus, pharmacological interventions, alkaline phosphatase, γ-glutamyl transpeptidase

## Abstract

**Background and objective:** Pruritus is a common complication in patients with primary biliary cholangitis (PBC). The pathogenesis is not clear, and also the precise therapeutic measures remain alluring. In order to systematically evaluate the efficacy and safety of drug interventions in the treatment of pruritus associated with PBC, this systemic review and meta-analysis was conducted.

**Methods:** The randomized controlled trials (RCTs) on drug interventions in the treatment of pruritus associated with primary cholangitis were searched in the electronic databases of PubMed, EMBASE, Cochrane Library, Web of Science, and ClinicalTrials.gov. Two researchers independently screened the literature, extracted and integrated the data, and assessed the bias risk of the selected literature, according to the *Cochrane handbook*. Finally, the STATA 15.0 software was used for the meta-analysis.

**Results:** A total of 23 RCTs involving 2,194 patients were studied, that included 12 pharmacological interventions. In terms of itching relief, compared with placebo, UDCA, methotrexate and GSK2330672 had a definite effect in improving pruritus (pruritus remission rate before and after treatment, *p <* 0.05). In terms of serum indexes, compared with placebo group, UDCA, OCA, rifampicin, cyclosporine, NGM282, seladelpar and colchicine may improve blood alkaline phosphatase (ALP) (*p <* 0.05), but only rifampicin showed low heterogeneity. UDCA, bezafibrate, OCA, rifampicin, NGM282 and others may improve blood γ-glutamyl transpeptidase (γ-GGT) (*p <* 0.05), but due to the high heterogeneity and the limitation of research samples, a clear conclusion cannot be drawn. In terms of adverse events, except high (>15 mg/kg/day) and low doses (<13 mg/kg/day) of UDCA increased the incidence of adverse events, there were no risk of increasing the incidence of adverse events compared with placebo (*p >* 0.05), and a moderate dose of UDCA (13–15 mg/kg/day) and malotilate (1,500 mg/day) may also help in reducing the incidence of adverse events (*p <* 0.05).

**Conclusion:** UDCA, methotrexate and GSK2330672 may relieve itching in patients with PBC, but there is a lack of robust evidence to support their effect on ALP or γ-GGT. Due to the heterogeneity in the published studies, based on the present review, we cannot explicitly recommend any specific drug for the treatment of PBC-related pruritus.

**Systematic Review Registration:** link-https://osf.io/2g8ya, identifier 10.17605/OSF.IO/2G8YA

## Introduction

Primary biliary cholangitis (PBC), formerly known as primary biliary cirrhosis, is an autoimmune liver disease, which is predominantly seen in women. The interaction of specific anti-mitochondrial antibodies with specific autoantigens, accompanied by pathophysiological processes such as bile duct injury, cholestasis, liver fibrosis and even liver cirrhosis, are the main features of the pathological progress of PBC (Lee et al., 2019; Gulamhusein and Hirschfield, 2020). At present, the incidence of PBC is geographically varied, and the number is on the rise too. According to a survey, there are 118.75 cases of prostate cancer per million people in the Asia-Pacific region (Zeng et al., 2019), 218.1 cases per million people in North America, 145.9 cases per million people in Europe, and 189.0 cases per million in Victoria, Australia (J et al., 2020). It was reported to be 346.0 cases per million in Sweden (HU et al., 2019), 149.0 cases per million in Slovakia (Drazilova et al., 2020) and, 279.0 cases per million in Italy (Rajaobelina et al., 2019). Environmental and genetic factors play an important role in the occurrence of the disease (Carey et al., 2015). The levels of serum alkaline phosphatase (ALP) and γ-glutamyl transpeptidase (γ-GGT) are generally increased in patients with PBC.

Fatigue and pruritus are the most common symptoms in patients with PBC. Scratching, sleep deprivation, depression and, even suicidal thoughts caused by itching affect 20%–70% of patients (Beuers et al., 2014; Carey et al., 2015), seriously affecting their quality of life. The accumulation and deposition of bile acid and bile salt, the regulation of lysophosphatidic acid, the abnormality of endogenous opioid receptors and the effects of serotonin and substance P are considered to be the main pathophysiological mechanisms of cholangitis pruritus (Bray et al., 2018). Even so, the pathogenesis of cholestatic pruritus has not been well described because of its diversity and complexity (Tajiri and Shimizu, 2017; SP et al., 2019).

A cross-sectional study of 2194 PBC pruritus patients in the United Kingdom shows that there is a lack of adequate understanding, management and guidelines for the disease, and there is insufficient evidence on the recommended treatment (Hegade et al., 2019). There exist few evidence-based guidelines for treating PBC pruritus, but the limitation is that there are specific clinical conditions where these guidelines cannot be applied. Ursodeoxycholic acid (UDCA) and OCA are drugs approved by the U.S. Food and Drug Administration (FDA) for the treatment of PBC, which can improve the liver biochemical indexes, prolong survival time and delay the development of esophageal varices. However, UDCA cannot completely cure this disease, and its effectiveness in the treatment of cholangitis pruritus is still controversial (JS et al., 2012). Interestingly, another potential anti-PBC drug, OCA, was reported to increase the risk of itching (Trauner et al., 2019). The search for an effective treatment for PBC-related pruritus interventions has never stopped. Newer pharmacological interventions have been reported such as cholestyramine (TB et al., 1961), rifampicin (CN and SG, 1988), sertraline (MJ et al., 2007), ondansetron (JW et al., 2005), maralixibat (Mayo et al., 2019), ileal apical sodium bile acid transporter (ASBT) inhibitors such as GSK2330672 (Hegade et al., 2017). In order to determine the efficacy and safety of the available drugs for the treatment of PBC-associated pruritus, we conducted a systematic review and meta-analysis of pharmacological interventions in PBC-related pruritus.

## Materials and methods

We followed a predetermined protocol and the principles of the Preferred Reporting Items for Systematic Reviews and Meta-Analyses (PRISMA) for this systemic review and meta-analysis (DG et al., 2009).

### Search strategy

To systematically evaluate the efficacy of interventions, we searched PubMed, EMBASE, Cochrane Library, Web of Science, and ClinicalTrials.gov from inception up to June 2021. The search strategy was implemented by an experienced medical librarian.

Strategies were selected using a combination of medical subject headings (MeSH) and text words, and search terms included “primary biliary cholangitis (cirrhosis),” “pruritus” or “pruritis” or “itching,” and “randomized controlled trial.” The language was limited to English, and the publication status was not restricted.

### Selection criteria

The inclusion criteria were as follows

(1) The study was a randomized controlled trial (RCT).
(2) The diagnosis of PBC was based on at least two of the following: the presence of anti-mitochondrial antibody (AMA), cholestasis with an elevation of ALP activity, histopathologic evidence of nonsuppurative cholangitis and destruction of small or medium-sized bile ducts (KD et al., 2019), and patients of primary biliary cholangitis with persistent pruritus (course of disease≥3 months).
(3) All pharmacological interventions related to the treatment of pruritus in PBC.
(4) Outcomes related to the efficacy and safety of pruritus in PBC.

### Exclusion criteria

(1) The interventions are not suitable (more than three interventions or the study interventions beyond our study).
(2) PBC associated with other underlying diseases.
(3) Unavailable data (there is no data we need in the study).
(4) Absence of a clear basic information about the study subjects.
(5) Duplicate publications.
(6) Studies with ambiguous diagnostic criteria.

### Study selection and data extraction

Two reviewers (Chenyi Xu and Xuelian Lv) independently extracted basic information about the articles (article title, first author, year of publication, sample size, country or region), trial design (participants, interventions, time span of the trial, follow-up time), clinical efficacy and adverse events.

Clinical efficacy measures included pruritus scores; 0–10 numerical rating scale (NRS), quality of life scale for primary biliary cirrhosis (cholangitis) (PBC-40), and 5-D itch scale (or the pruritus relief rate before and after treatment). Secondary outcomes were laboratory parameters such as the changes in serum alkaline phosphatase (ALP) and gamma-glutamyl transpeptidase (γ–GGT). We considered both iatrogenic and non-iatrogenic adverse events, and carried out a quantitative analysis of these events.

### Risk of bias assessment

Two investigators evaluated each of the included RCTs and recorded the following six items, as per the *Cochrane Handbook for Systematic Reviews of Interventions* (Higgins and Green, 2008): the methods of blinding, the generation of information, data, distribution of randomized control sequences, selective reports and other possible problems. The risk of biases was marked as high, uncertain, or low.

### Statistical analysis

STATA15.0 (STATA statistical software: Release 15.0 College Station, TX: Stata Corp LP) was used to analyze the data. Successive mean differences in pruritus scores were reported as standardized mean differences (SMD), and binary variables used risk ratios (RR) or odds ratio (OR), providing a 95% confidence interval (CI) for each effect. Heterogeneity was evaluated by *I* ^2^, *p < 0.05* was considered to be statistically significant. The studies with high heterogeneity *(p ≤* 0.10 and *I* ^*2*^ *≥* 50%) were analyzed by random effect model, the studies with low heterogeneity (*p >* 0.10 and *I* ^*2*^ *<* 50%) were analyzed by fixed effect model. The stability of the results was evaluated by subgroup analysis and sensitivity analysis. If enough studies were included in meta-analysis (*n ≥* 10), funnel chart analysis was used for evaluate publication bias.

## Results

### Study selection

**FIGURE 1:**
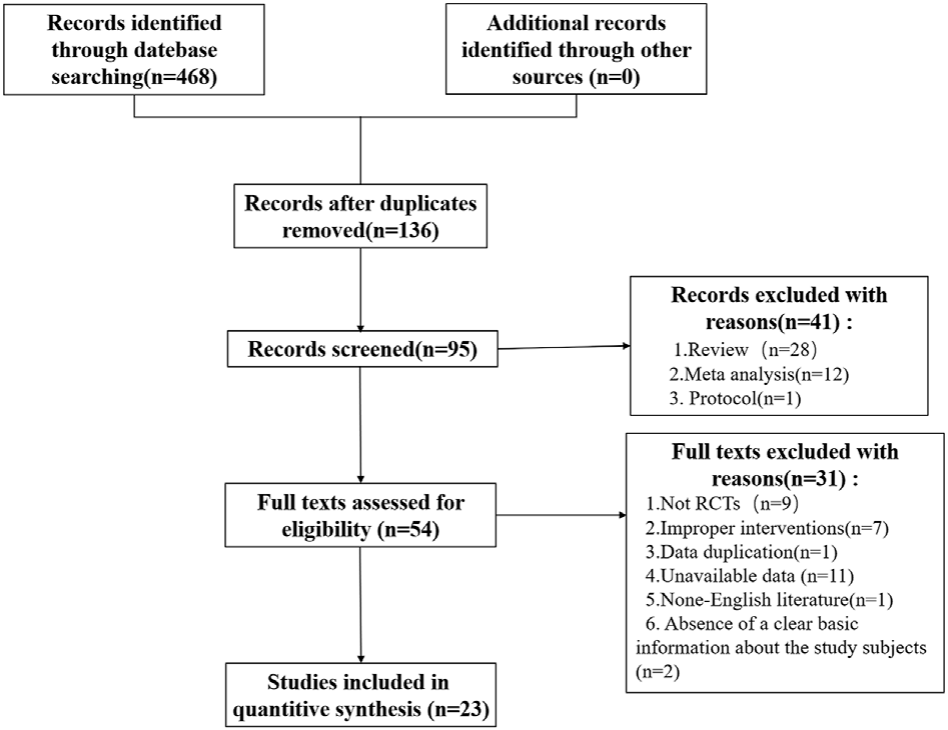
Flow chart of trail selection.

A total of 468 records were retrieved from the electronic database (PubMed *n* = 90, EMBASE *n* = 80, Cochrane Library *n* = 151, Web of Science *n* = 106, ClinicalTrials.gov *n* = 41), and 332 records were excluded after duplicates removed. After browsing the titles and abstracts, 41 articles were excluded based on article type (review *n* = 28, meta-analysis *n* = 12, protocol *n* = 1). After that, 54 articles were assessed as full text and 31 were excluded for the following reasons: not RCTs (*n* = 9), improper intervention (*n* = 7), data duplication (*n* = 1), unavailable data (*n* = 11), non-English literature (*n* = 1), absence of a clear basic information about the study subjects (*n* = 2). Finally, 23 studies were obtained. The details are shown in the flowchart (Figure 1). Of the 23 studies included, eight were RCTs studies of UDCA (Poupon et al., 1990; Oka et al., 1991; Poupon et al., 1991; Battezzati et al., 1993; Lindor et al., 1994; Heathcote et al., 1995; Vuoristo et al., 1995; Parés et al., 2000). The remaining 15 RCTs contained three studies of obeticholic acid in PBC (Hirschfield et al., 2015; Nevens et al., 2016; Kowdley et al., 2018), two studies of bezafibrate in PBC (Kanda et al., 2003; Corpechot et al., 2018), and two studies of rifampicin (Bachs et al., 1989; Podesta et al., 1991), and one RCT each evaluating the ileal bile acid transporter inhibitor GSK2330672 (Hegade et al., 2017), FGF19 analog NGM282 (Mayo et al., 2018), selective PPAR-δ agonist seladelpar (MBX-8025) (Jones et al., 2017), cyclosporine (Wiesner et al., 1990), colchicine (Almasio et al., 2000), methotrexate (Hendrickse et al., 1999), maralixibat (Mayo et al., 2019) and malotilate (Listed, 1993).

The 23 RCTs included 2,194 patients, 92% of the study population was women, and the average age was 54.9 years. The studies included in our systematic review are shown in Table 1.

**TABLE 1:** Basic characteristics of 23 included studies.

Study and year	Design	Population	Sample size	Intervention	Outcomes	Follow-up	Concomitant treatment
***UDCA***							
Hiroshi OKA 1990	RCT	PBC. Mean age 59 years, 89% female	22 UDCA 23 Placebo	UDCA 8–12 mg/kg/day	(1) (2) (3) (4)	2 years	None detailed
Poupon RE 1991	RCT	PBC. Mean age 56 years, 92% female	73 UDCA 73 Placebo	UDCA 13–15 mg/kg/day	(1) (2) (3) (4)	2 years	None detailed
Matti 1995	RCT	PBC. Mean age 54.5 years, 92% female	30 UDCA 31 Placebo	UDCA 12–15 mg/kg/day	(1) (2) (3) (4)	2 years	None detailed
Albert Parés 2000	RCT	PBC. Mean age 54.1 years, 93% female	99 UDCA 93 Placebo	UDCA 14–16 mg/kg/day	(1) (2) (4)	2 years	Cholestyramine at least 2 h after the intake of UDCA or Placebo
E.Jenny heathcote 1994	RCT	PBC.Mean age 56.4 years, 93% female	111 UDCA 111 Placebo	UDCA 14 mg/kg/day	(1) (3) (4)	2 years	Cholestyramine, in the morning or at least 4 h before the trial capsules
P.M.Battezzati1993	RCT	PBC.Mean age 54.5 years, 89% female	44 UDCA 44 Placebo	UDCA 8.7 mg/kg/day	(1) (3) (4)	1 year	Cholestyramine take at least 4 h before or after the study drug
Poupon RE 1990	RCT	PBC.Mean age 56.5 years, 91% female	70 UDCA 68 Placebo	UDCA 13–15 mg/kg/day	(1) (2) (3) (4)	2 years	None detailed
K. D. Lindor1994	RCT	PBC.Mean age 53 years, 89% female	89 UDCA 91Placebo	UDCA 13–15 mg/kg/day	(1) (3) (4)	2 years	Cholestyramine were asked to take drug 2 h after their study drug
Study and year	Design	Population	Sample size	Intervention	Outcomes	Follow-up	Concomitant treatment
***Rifampicin***							
Laura Bachs 1989	RCT	PBC. Mean age 49.7 years, 100% female	21 Rifampicin	Rifampicin 10 mg/kg/day, Phenobarbitone 3 mg/kg	(1) (2) (3) (4)	14 days	None detailed
			18 Phenobarbitone				
Ana Podesta 1991	RCT	PBC. Mean age 43 years, 93% female	14 Rifampicin	Rifampicin 300 mg twice a day	(1) (3) (4)	3 months	None detailed
			14 Placebo				
***GSK2330672***							
Vinod S Hegade 2017	RCT	PBC. Mean age 52.9 years, 90% female	22 Placebo run-in	GSK2330672	(1) (2) (3) (4)	28 days	19 patients taking UDCA (14 mg/kg/day) during study period
			21 GSK2330672	45 mg twice per day on days 1–3, and 90 mg twice daily on days4–14			
			21 Placebo				
***Maralixibat***							
M. J. Mayo 2019	RCT	PBC. Mean age 53.4 years, 91% female	21 Maralixibat 10 mg	Maralixibat 10 mg,10 mg/day	(1) (2) (3) (4)	13 weeks	None detailed
			21 Maralixibat 20 mg	Maralixibat 20 mg, 20 mg/day			
			24 Placebo				
Study and year	Design	Population	Sample size	Intervention	Outcomes	Follow-up	Concomitant treatment
***Obeticholic Acid***							
F. Nevens 2016	RCT	PBC. Mean age 56 years, 91% female	70 OCA5-10 mg	OCA 5–10 mg/day or 10 mg/day	(1) (3)	1 year	standard-of-care UDCA (13–15 mg/kg/day) or not
			73 OCA 10 mg				
			73 Placebo				
Kris V. Kowdley 2018	RCT	PBC. Mean age 54 years, 85% female	20 OCA 10 mg	OCA10 mg/day or 50 mg/day	(1) (3) (4)	3 months	None detailed
			16 OCA 50 mg				
			23 Placebo				
G. M Hirschfield 2014	RCT	PBC. Mean age 55.1 years, 95% female	38 OCA 10 mg	OCA10 mg/day or 25 mg/day or 50 mg/day	(2) (3) (4)	3 months	UDCA (15.6–16.3 mg/kg/day)
			48 OCA 25 mg				
			41 OCA 50 mg				
			38 Placebo				
***Bezafibrate***							
C. Corpechot 2018	RCT	PBC.Mean age 53 years, 95% female	50 Bezafibrate	Bezafibrate 400 mg/day	(1) (2) (3) (4)	2 years	UDCA (13–15 mg/kg/day)
			50 Placebo				
Tatsuo Kanda 2003	RCT	PBC.Mean age 56 years, 86% female	11 UDCA with Bezafibrate	Bezafibrate 400 mg/day	(1) (2) (3) (4)	0.5 years	UDCA (600 mg/day)
			11 UDCA				
Study and year	Design	Population	Sample size	Intervention	Outcomes	Follow-up	Concomitant treatment
***NGM282***							
M. J. mayo 2018	RCT	PBC. Mean age 56.3 years, 91% female	14 NGM282 0.3 mg	NGM282 0.3mg, 0.3 mg/day	(1) (2) (3) (4)	28 days	None detailed
			16 NGM282 3 mg	NGM282 3mg, 3 mg/day			
			15 Placebo				
***Seladelpar (MBX-8025)***							
David Jones 2017	RCT	PBC. Mean age 56 years, 95% female	13 Seladelpar50 mg	seladelpar50 mg,50 mg/day	(1) (2) (3) (4)	12 weeks	Continue UDCA at the same dose
			13Seladelpar200 mg	Seladelpar200 mg,200 mg/day			
			12 Placebo				
***Malotilate***							
A European Multi--centre Study Group 1993	RCT	PBC. Mean age 54.4 years, 96% female	52 Malotilate	Malotilate:500 mg, three times a day	(1) (2) (3) (4)	1.5 years	None detailed
			49 Placebo				
***Cyclosporine***							
R.H.Wiesner 1990	RCT	PBC. Mean age 46.8 years, 97% female	19 Cyclosporine	cyclosporine	(1) (2) (3) (4)	2 years	None detailed
			10 Placebo	4 mg/kg/day			
Study and year	Design	Population	Sample size	Intervention	Outcomes	Follow-up	Concomitant treatment
***Colchicine***							
P. L. Almasio 2000	RCT	PBC. Mean age 54.4 years, 90% female	46 Colchicine plus UDCA	Colchicine, 1 mg/daily	(1) (2) (3) (4)	3 years	Cholestyramine, no more than 8 g/day
			44 UDCA	UDCA (5.4–11.6 mg/kg/day)			
***Methotrexate***							
M.T. Hendrickse1999	RCT	PBC. Mean age 57 years, 92% female	30 MTX	MTX, 7.5 mg/wk (on Friday, Saturday, and Sunday of each week, 2.5 mg/day)	(1) (2) (3) (4)	6 years	None detailed
			30 Placebo				

Abbreviations: PBC, Primary biliary cholangitis; MTX, Methotrexate; OCA, Obeticholic Acid; UDCA, Ursodeoxycholic acid; (1) ALP, Alkaline phosphatase; (2) γ-GGT, Gamma-glutamyltranspeptidase; (3)Number with pruritus, grade of pruritus; (4)Adverse event rate(%).

### Study quality

**FIGURE 2:**
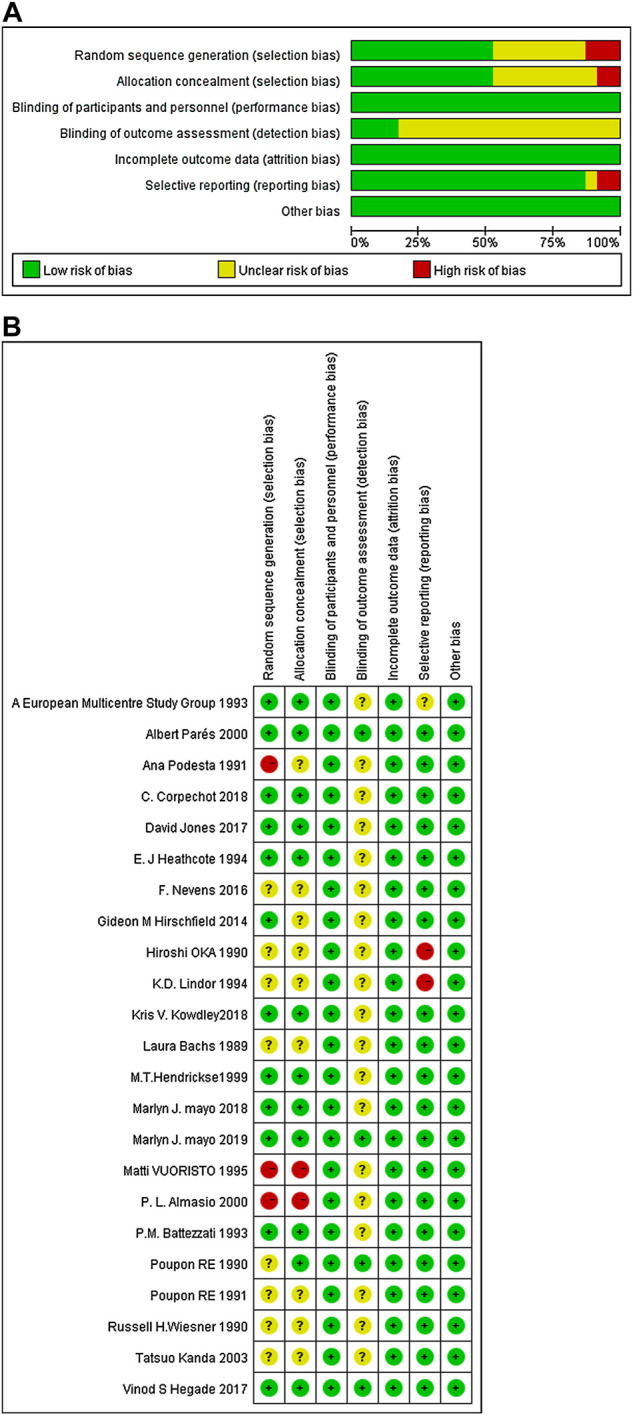
The quality assessment of included trials based on the Cochrane risk assessment tool. **(A)** Each risk of bias item presented as percentages across all included studies; **(B)** Each risk of bias item for each included study.

Of all the 23, 11 studies mentioned the randomization techniques (including random sampling with computer (Battezzati et al., 1993; Hendrickse et al., 1999; Hirschfield et al., 2015; Hegade et al., 2017; Jones et al., 2017; Corpechot et al., 2018; Kowdley et al., 2018; Mayo et al., 2019), or interactive voice/web response system (Mayo et al., 2018), or random numbers (Listed, 1993; Heathcote et al., 1995)), while two studies were considered “high risk” because the random methods were inappropriate (Podesta et al., 1991; Vuoristo et al., 1995). Twelve studies followed allocation concealment (including third-party random allocation (Poupon et al., 1990; Battezzati et al., 1993; Lindor et al., 1994; Jones et al., 2017; Corpechot et al., 2018; Mayo et al., 2018; Mayo et al., 2019), sequence number (Listed, 1993; Heathcote et al., 1995; Hendrickse et al., 1999; Hegade et al., 2017), or envelope (Parés et al., 2000)). Of all the included studies, two followed single-blind (Oka et al., 1991; Heathcote et al., 1995), and the rest followed double-blind designs. The data evaluator was blinded in five studies. All the 23 studies reported complete data and there was no risk of other biases. The Cochrane deviation risk assessment tool was used for risk assessment, and the results are shown in Figures 2A,B.

### Pruritus (relief rate)

**FIGURE 3:**
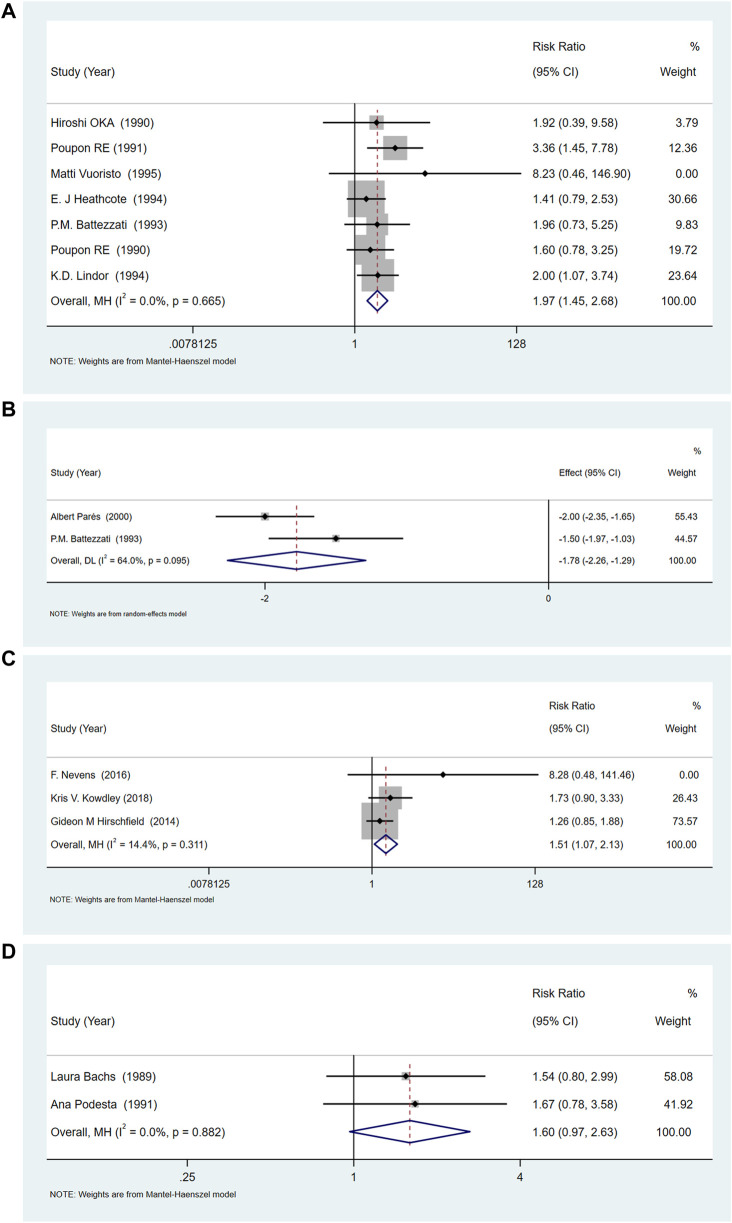
**(A)**The effect of UDCA on pruritus relief compared with placebo. **(B)** Two studies of UDCA in pruritus scores compared with placebo. **(C)** OCA increases the risk of pruritus compared with placebo. **(D)** The effect of Rifampicin on the rate of pruritus relief compared with placebo.

Most trials reported only the number of patients with pruritus pre-treatment and post-treatment, so we considered the relief rate as the primary outcome. Among the eight studies (Poupon et al., 1990; Oka et al., 1991; Poupon et al., 1991; Battezzati et al., 1993; Lindor et al., 1994; Heathcote et al., 1995; Vuoristo et al., 1995; Parés et al., 2000), of UDCA seven documented itching. The results showed that UDCA combined with or without cholestyramine, compared to placebo (RR = 1.85, 95%CI (1.40, 2.45), *p <* 0.001, *I* ^*2*^ = 0.0%), had a significant difference in relieving pruritus (Figure 3A). Among these, two studies recorded the itching score in detail (Battezzati et al., 1993; Parés et al., 2000). Compared with placebo, UDCA combined with cholestyramine significantly reduced the itching score (SMD = −1.78, 95% CI (−2.26, −1.29), *p <* 0.001, *I* ^*2*^ = 64%), but had a high heterogeneity (*p =* 0.095, *I* ^*2*^ = 64.0%) (Figure 3B).

Three studies (Hirschfield et al., 2015; Nevens et al., 2016; Kowdley et al., 2018) compared the relief of pruritus after OCA treatment, and the heterogeneity among studies was low (*p =* 0.311*, I* ^*2*^ = 14.4%). Meta-analysis showed that OCA increased the incidence of pruritus, compared with placebo (RR = 1.511, 95%CI (1.07, 2.12), *p =* 0.018) (Figure 3C).

Two studies (Bachs et al., 1989; Podesta et al., 1991) compared the relief of pruritus after rifampicin treatment, and the heterogeneity between the studies was low (*p =* 0.882, *I* ^*2*^ = 0.0%). The results of meta-analysis showed that rifampicin had no significant effect on itching compared with placebo or control drugs [RR = 1.595, 95%CI (0.97, 2.63), *p* = 0.067] (Figure 3D).

One study (Wiesner et al., 1990) reported the incidence of pruritus in patients with PBC, before and after treatment, with cyclosporine and placebo. The results showed that cyclosporine did not significantly reduce the incidence of pruritus compared with placebo [RR = 0.726, 95%CI (0.51, 1.03), *P* = 0.072]

One study (Mayo et al., 2018) reported a comparison between NGM282 and placebo. The results suggested that NGM282 had no significant effect on 5-D itch score [SMD = −0.429, 95%CI (−1.06, 0.20), *p* = 0.179] and VAS score [SMD = 0.335, 95%CI (−0.29, 0.96), *p* = 0.293] in patients with PBC.

One study (Listed, 1993) reported a comparison between malotilate and placebo, which suggested that malotilate did not significantly reduce the incidence of pruritus [RR = 1.083, 95%CI (0.43, 2.73), *p* = 0.865].

One study (Jones et al., 2017) reported the comparison of seladelpar and placebo after treatment. The results suggested that seladelpar did not significantly reduce pruritus in patients with PBC. The 5-D itch score [SMD = 0.000, 95%CI (−0.79, 0.79), *p* = 1.000] and VAS score [SMD = 0.138, 95%CI(−0.65, 0.92), *p* = 0.731] showed no significant alteration.

One study (Mayo et al., 2019) reported a comparison between maralixibat and placebo. The results suggested that maralixibat did not significantly reduce the pruritus 5-D itch score in patients with PBC [SMD = −0.09, 95%CI (−0.59, 0.41), *p* = 0.725].

A study (Hendrickse et al., 1999) on methotrexate showed that, compared with placebo, it significantly reduced pruritus scores [SMD = 1.000, 95%CI (−1.54, −0.46), *p* = 0.000].

In a comparative study (Vuoristo et al., 1995) between colchicine plus UDCA and UDCA, it was found that colchicine had no significant effect on reducing the incidence of pruritus [RR = 0.964, 95%CI (0.42, 2.24), *p* = 0.931].

Another study (Hegade et al., 2017) on GSK2330672 used different scoring systems to evaluate the change in pruritus score before and after treatment. The percentage changes from baseline itch scores were -57% [95% CI (−73, −42), *p < 0.0001*] in NRS, −31% (−42∼-20, *p < 0.0001*) in PBC-40 itching, and −35% (−45∼−25, *p < 0.0001*) in 5-D itch score.

One study (Parés et al., 2000) of UDCA could not be analyzed because of the lack of data. Indicators changes before and after treatment for all included studies are listed in Table 2.

**TABLE 2:** Summary of results for studies were not included in the meta-analysis.

Study ID	Intervention	Change in pruritus (event/noevent)	Change in ALP(U/L) (MD ± SD)	Change in γ-GGT (U/L) (MD ± SD)	Chang in adverse events (event/noevent)
F. Nevens 2016	OCA 10 mg	6/50	−130 ± 15	Not reported	Not reported
	Placebo	−19/28	−14 ± 15		
K.V. Kowdley 2018	OCA 10 mg	Not reported	−159 ± 67	Not reported	33/3
	Placebo		1 ± 17		21/2
Hirschfield 2014	OCA 10 mg	Not reported	Not reported	−92 ± 31	34/4
	Placebo			−1 ± 31	32/6
C. Corpechot 2018	Bezafibrate	Not reported	−60 ± 4	−38 ± 9	43/7
	Placebo		0 ± 9	7 ± 16	45/5
Tatsuo Kanda 2003	Bezafibrate + UDCA	1/6	−310 ± 234	−31 ± 20	1/10
	UDCA	0/5	−5 ± 219	−1 ± 14	0/11
Poupon RE 1990	UDCA	18/20	−417 ± 403	−413 ± 394	Not reported
	Placebo	10/20	20 ± 328	−32 ± 512	
Albert Parés 2000	UDCA	Not reported	−466 ± 56	−256 ± 33	9/90
	Placebo		−97 ± 64	−17 ± 30	6/87
P.M.Battezzati1993	UDCA	11/29	Not reported	Not reported	4/40
	Placebo	5/34			1/43
E.J Heathcote 1994	UDCA	24/63	−42 ± 99	Not reported	12/99
	Placebo	16/63	3 ± 98		19/92
K. D. Lindor1994	UDCA	27/21	−711 ± 811	Not reported	21/68
	Placebo	12/39	−300 ± 633		43/48
Laura Bachs 1989	Rifampicin	19/2	−316 ± 647	−99 ± 187	1/20
	Phenobarbitone	8/10	125 ± 795	173 ± 294	3/15
Study ID	Intervention	Change in pruritus (event/noevent)	Change in ALP (U/L) (MD ± SD)	Change in γ-GGT (U/L) (MD ± SD)	Chang in Adverse events (event/noevent)
Ana Podesta 1991	Rifampicin	14/0	−383 ± 683	Not reported	0/14
	Placebo	6/8	−92 ± 697		0/14
M. J. mayo 2018	NGM282 3 mg	5-D itch:-2.1 ± 4.7, VAS:-6.2 ± 15.6	−66 ± 57.7	−50.8 ± 71.9	22/8
	Placebo	5-D itch:-0.2 ± 3.8, VAS:-12.6 ± 24.9	3.3 ± 57.3	−5.6 ± 47.3	12/3
R. H.Wiesner 1990	Cyclosporine	16/1	−143 ± 623	Not reported	15/4
	Placebo	3/6	273 ± 345		5/5
A European Multi--centre study group	Malotilate	8/16	−61 ± 111	Not reported	13/39
	Placebo	6/20	−30 ± 150		2/47
David Jones 2017	Seladelpar200 mg (continue UDCA)	4/1	−156 ± 100	Not reported	12/12
	Placebo (continue UDCA)	3/1	4.7 ± 3.5		6/7
M.J. mayo 2019		5-D itch score			
	Maralixibat 10 mg	−6.9 ± 6.8	−7.4 ± 80.4	28.6 ± 249.8	15/6
	Placebo	−6.3 ± 6.4	7.3 ± 80.0	45 ± 231	11/13
M.T.Hendrickse 1999		Pruritus scores	Not reported	Not reported	
	Methotrexate	−0.1 ± 0.2			21/9
	Placebo	0.1 ± 0.2			19/11
P. L. Almasio 2000	Colchicine + UDCA	9/16	Not reported	Not reported	6/40
	UDCA	9/18			11/33
V.S Hegade 2017		5-D itch scale	Not reported	Not reported	
	GSK2330672	6.5 ± 22.0			17/4
	Placebo	—			17/4

Abbreviations: MD, Mean Deviation; SD, Standard Deviation; VAS, Visual Analogue Score; Ursodeoxycholic acid; ALP, Alkaline phosphatase; γ-GGT, Gamma-glutamyltranspeptidase.

### Alkaline phosphatase

**FIGURE 4:**
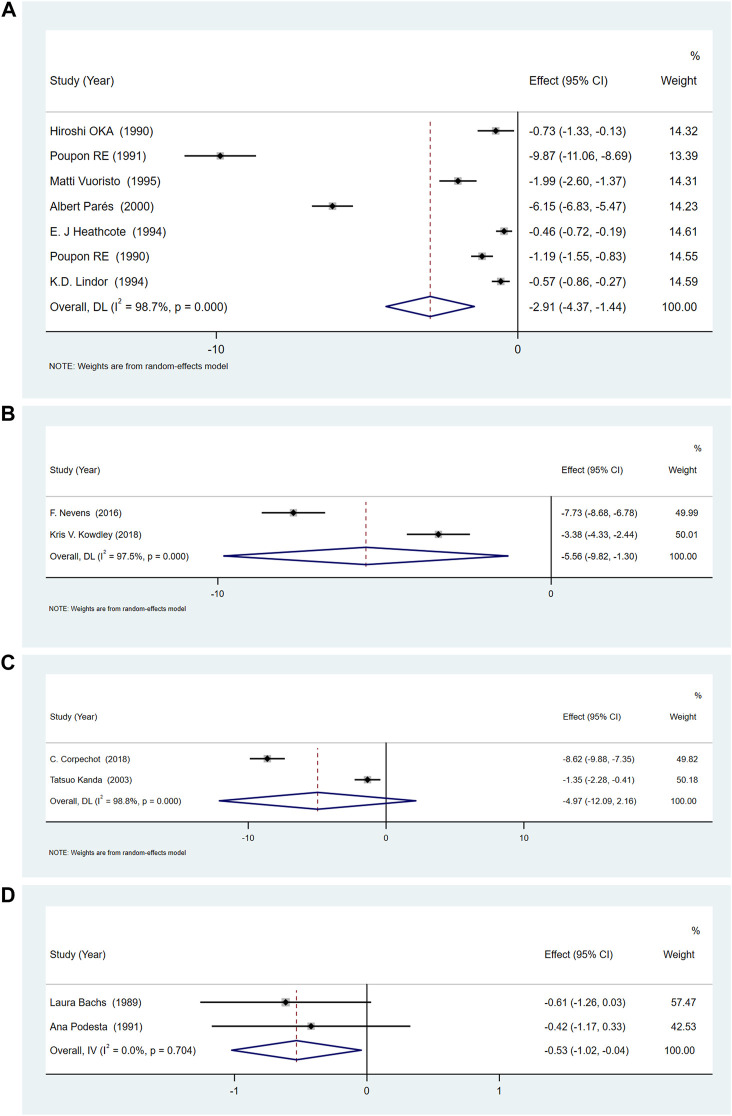
**(A)**The effect of UDCA in serum ALP. **(B)** The effect of OCA on serum ALP. **(C)** The effect of Bezafibrate on serum ALP. **(D)** The effect of Rifampicin on serum ALP.

Compared with placebo, UDCA, OCA and rifampicin could reduce serum ALP levels, UDCA [SMD = -2.91, 95%CI (−4.37, −1.44), *p* = 0.000], OCA [SMD = −5.56, 95%CI (−9.82, −1.30), *p* = 0.011], rifampicin [SMD = −0.53, 95%CI (−1.02, −0.04), *p* = 0.033]. However, the change brought about by bezafibrate [SMD = −4.98, 95%CI (−12.09, 2.16), *p* = 0.172] was not statistically significant. But the ALP results showed a high degree of heterogeneity in UDCA (*p* = 0.000, *I* ^*2*^ = 98.7%), OCA (*p* < 0.01, *I* ^*2*^ = 97.5%), and bezafibrate (*p* < 0.01, *I* ^*2*^ = 98.8%) (Figures 4A–C). However, the heterogeneity of the results of rifampicin was low (*p* = 0.704, *I* ^*2*^ = 0.0%) (Figure 4D). We conducted sensitivity analysis (Supplementary Material S1) and subgroup analysis (according to UDCA dose, low: <13 *mg/kg/d,* medium: 13–15 *mg/kg/d*, high:>15 *mg/kg/d*) (Supplementary Material S2), study area (Asia, Europe, America) (Supplementary Material S3), and if cholestyramine was used as a combination (Supplementary Material S4).

A study (Wiesner et al., 1990) comparing cyclosporine with placebo found that, cyclosporine was superior to placebo in reducing ALP [SMD = −5.36, 95%CI (−6.98, −3.74), *p* = 0.000].

One study (Mayo et al., 2018) reported a comparison between NGM282 and placebo. The results suggested that NGM282 could significantly reduce the level of ALP in patients with PBC [SMD = −1.205, 95%CI (−1.98, −0.44), *p* = 0.002].

One study (Listed, 1993) reported a comparison between malotilate and placebo. The results showed no significant difference in the reduction of ALP between the two [SMD = −0.236, 95%CI (−0.63, 0.16), *p* = 0.238].

One study (Jones et al., 2017) reported a comparison between seladelpar and placebo, which suggested that seladelpar significantly reduced ALP [SMD = −2.224, 95%CI(−3.24, −1.21], *p* = 0.000).

One study (Mayo et al., 2019) on comparison between maralixibat and placebo concluded that maralixibat did not significantly reduce serum ALP [SMD = −0.183, 95%CI (−0.77, 0.40), *p* = 0.540].

A study (Vuoristo et al., 1995) on colchicine plus UDCA against UDCA alone showed that, compared with UDCA, colchicine plus UDCA significantly reduced ALP [SMD = −0.183, 95%CI (−0.77, 0.40), *p* = 0.540]. The other studies on methotrexate (Listed, 1993), colchicine (Almasio et al., 2000)and GSK2330672 (Hegade et al., 2017) did not report any change of ALP level after drug treatment.

### Gamma-glutamyl transpeptidase

**FIGURE 5:**
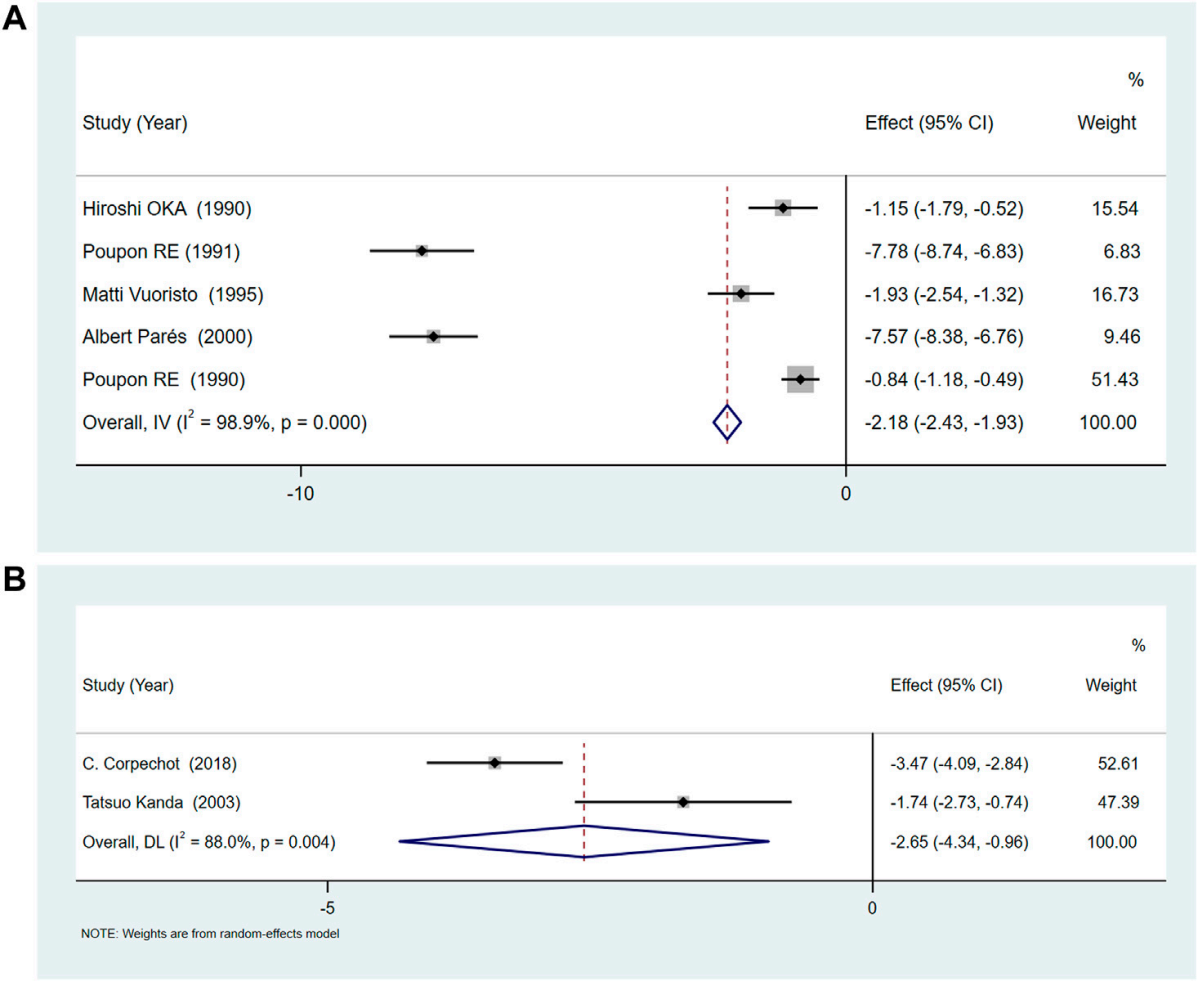
**(A)**The effect of UDCA on serum γ-GGT. **(B)** The effect of Bezafibrate on serum γ-GGT.

Blood γ-GGT levels were reported in 5 RCTs of UDCA (Poupon et al., 1990; Oka et al., 1991; Poupon et al., 1991; Vuoristo et al., 1995; Parés et al., 2000)and two of bezafibrate (Kanda et al., 2003; Corpechot et al., 2018). The results showed that UDCA and bezafibrate could reduce serum γ-GGT levels (UDCA [SMD = −2.18, 95%CI (−2.43, −1.93), *p* = 0.000], bezafibrate [SMD = −2.65, 95%CI (−4.34, 0.96), *p* = 0.002]) (Figures 5A,B). However, there was obvious heterogeneity for UDCA (*p* < 0.01, *I* ^*2*^ = 98.9%) and bezafibrate (*p <* 0.01, *I* ^*2*^ = 88.8%). Therefore, we conducted a subgroup analysis based on the dose of UDCA (Supplementary Material S5), study area (Supplementary Material S6) and whether cholestyramine was combined with UDCA (Supplementary Material S7), to evaluate its effect on serum γ-GGT. Sensitivity analysis showed that the results were consistent (Supplementary Material S8).

A study (Hirschfield et al., 2015) on the effect of OCA in serum γ-GGT showed that OCA reduced serum γ-GGT than placebo [SMD = −2.935, 95%CI (−3.59, −2.28), *p* = 0.000].

One study (Bachs et al., 1989) reported a comparison between rifampicin and placebo. The results suggested that the former could significantly reduce the level of serum γ-GGT [SMD = −1.123, 95%CI (−1.80, −0.44), *p* = 0.001].

One study (Mayo et al., 2018) reported a comparison between NGM282 and placebo. The results suggested that NGM282 can reduce the level of γ-GGT [SMD = −0.738, 95%CI (−1.47, −0.01), *p* = 0.047] in patients with PBC.

A study (Mayo et al., 2019) on the effect of maralixibat on serum γ-GGT, compared with placebo, showed no significant difference between the two drugs [SMD = −0.068, 95% CI (−0.65, 0.52), *p* = 0.819].

The other two studies of OCA (Nevens et al., 2016; Kowdley et al., 2018), three of UDCA (Battezzati et al., 1993; Lindor et al., 1994; Heathcote et al., 1995), one of rifampicin (Podesta et al., 1991), one of cyclosporine (Wiesner et al., 1990), one of malotilate (Listed, 1993), one of seladelpar (Jones et al., 2017), one of methotrexate (Hendrickse et al., 1999), one of colchicine (Almasio et al., 2000) and one of GSK2330672 (Hegade et al., 2017) did not report any change of serum γ-GGT level after treatment.

### Adverse events

**FIGURE 6:**
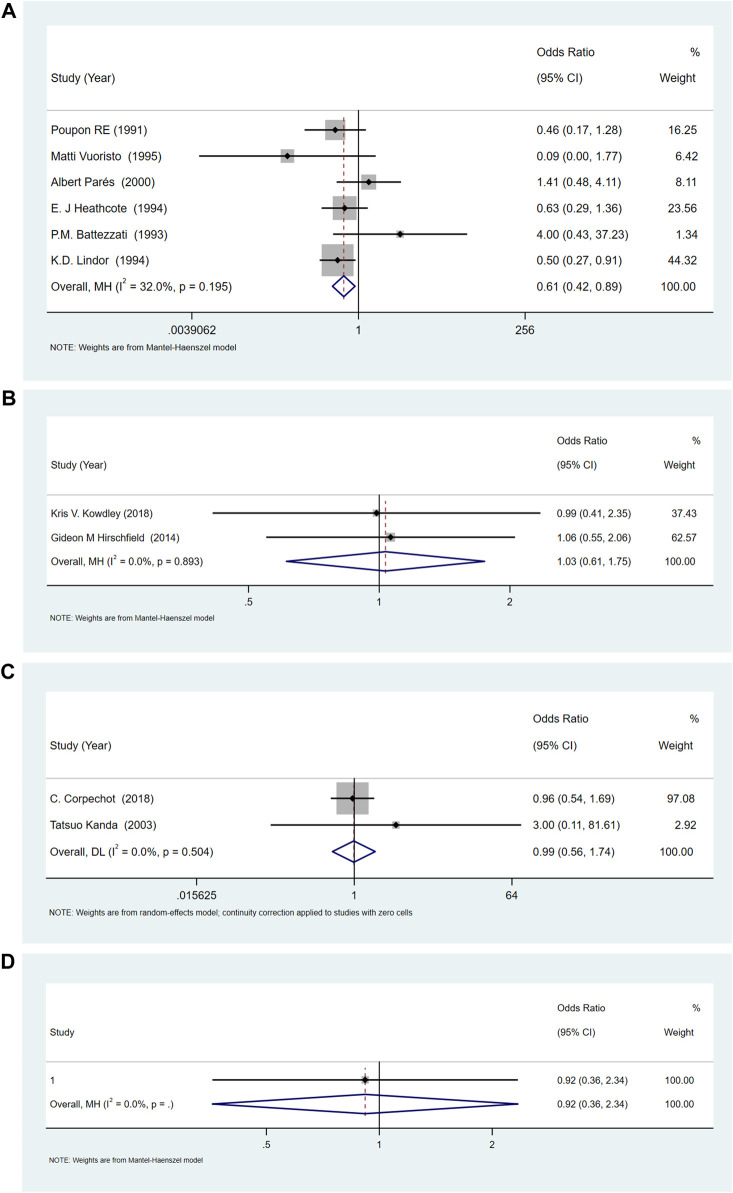
**(A)**The adverse events for UDCA. **(B)** The adverse events for OCA. **(C)** The adverse events for Bezafibrate. **(D)** The adverse events for NGM282.

Compared with placebo, the incidences of adverse events with UDCA were lower [OR = 0.61, 95%CI (0.42, 0.89), *p* = 0.011], and there was no significant difference in OCA (OR = 1.03, 95%CI (0.61, 1.75), *p* = 0.901) and bezafibrate (OR = 0.99, 95%CI (0.56, 1.74), *p* = 0.967). The results showed that the heterogeneity was low, (for UDCA: *p* = 0.195 and *I* ^*2*^ = 32.0%, for OCA: *p* = 0.892 and *I* ^*2*^ = 0.0%, and for bezafibrate: *p* = 0.504, *I* ^*2*^ = 0.0%) (Figures 6A–C).

Sensitivity analysis of UDCA indicated that the results were consistent (Supplementary Material S9). Subgroup analysis based on UDCA dose (Supplementary Material S10), study area (Supplementary Material S11), year of publication (Supplementary Material S12) and whether UDCA was combined with cholestyramine (Supplementary Material S13), showed that the occurrence of adverse events was dose-dependent. Both high (>15 *mg/kg/day*) and low doses (<13 *mg/kg/day*) of UDCA increased the incidence of adverse events, while the middle dose (13–15 *mg/kg/day*) of UDCA did not increase the incidence of adverse events.

A study (Mayo et al., 2018) on the comparison of adverse reactions between NGM282 and placebo showed no significant difference [OR = 0.917, 95%CI (0.36, 2.34), *p* = 0.856].

One study (Wiesner et al., 1990) reported no significant difference in the incidence of adverse events when cyclosporine and placebo were compared [OR = 1.579, 95%CI (0.44, 5.62), *p* = 0.481].

A study (Listed, 1993) compared malotilate with placebo, showed that malotilate was superior than placebo in reducing adverse events [OR = 6.125, 95%CI (1.31, 28.52), *p* = 0.021].

Two separate studies (Jones et al., 2017; Mayo et al., 2019) reported no significant difference in the reduction of adverse events between seladelpar (MBX-8025) and placebo groups [OR = 1.820, 95%CI (0.59, 5.62), *p* = 0.298], and between Maralixibat and placebo [OR = 1.558, 95%CI (0.59, 4.13), *p* = 0.372].

Similarly, studies (Hendrickse et al., 1999; Almasio et al., 2000; Hegade et al., 2017) reported no significant differences for the adverse events between methotrexate and placebo [OR = 1.105, 95%CI (0.50, 2.46), *p* = 0.806], colchicine and placebo [OR = 0.522, 95%CI (0.18, 1.53), *p* = 0.236], and GSK2330672 and placebo [OR = 1.000, 95%CI (0.41, 2.47), *p* = 1.000].

Two studies (Bachs et al., 1989; Podesta et al., 1991) reported no significant difference in the reduction of adverse events between rifampicin and placebo [OR = 0.286, 95%CI (0.03, 2.99), *p* = 0.296].

## Discussion

The rising incidence of pruritus in PBC and the lack of effective treatment methods were serious concerns. One study found that the incidence of pruritus can growing from 19% at the beginning to 80% in 10 years later (Prince et al., 2002). Pruritus in PBC was belong to cholestatic pruritus. Unlike histamine-related pruritus, cholestatic pruritus had no evidence-based guidelines because the underlying pathogenesis was unclear. Some studies had shown that itching symptoms often occured in female patients with PBC, accompanied by increasing levels of ALP and γ-GGT, but the mechanism behind it was still unknown. Existing studies had found that PBC-related pruritus was mainly related to bile acids, lysophosphatidic acid A (LPA), G-protein coupled bile acid receptor1 (GPBAR1), endogenous opioids, 5-hydroxytryptamine (5-HT), nitric oxide and substance P. But since the circulation level of these substances was not well correlated with the severity of pruritus, there may be complex interactions among multiple pruritus substances (Quarneti et al., 2015). Firstly, for the treatment of PBC-related pruritus, we need to rule out renal failure, psoriasis, idiopathic dermatitis and other diseases that can cause pruritus. Secondly, drugs treatment and non-drugs treatment measures should be adopted according to the severity of the disease. The first-line treatment drugs were mainly bile acid-binding resin (Gideon et al., 2017), such as colesevelam hydrochloride, which has better therapeutic effect than cholestyramine. Second-line therapeutic drugs such as rifampicin, opioid antagonists (naltrexone) and modulators of 5-HT receptor pathway (sertraline) can be used as supplements to first-line drugs. In addition, most of the non-drug treatments belong to invasive treatment strategies, and liver transplantation was the last choice for patients with intractable pruritus when all treatment strategies were ineffective. Unfortunately, due to lack of evidence-based evidence, the role of many drug interventions in the treatment of cholestatic pruritus, including PBC was uncertain. This result has also been confirmed in a recent related study (Dervout et al., 2022).

Moreover, drugs for PBC were indispensable in the treatment of pruritus. In recent years, newer target drugs were introduced, which mainly focus on reducing cholestasis and reduced bile acid toxicity, and are immunomodulatory in action and antifibrotic in nature (Shah and Kowdley, 2020). However, the efficacy of these drugs remains to be further evaluated. For example, OCA was a semisynthetic chenodeoxycholic acid analogue, which can inhibit bile acid synthesis and stimulate bile secretion to protect hepatocytes by activating farnesol X receptor. Amazingly, it was found that pruritus increased dose-dependently with OCA treatment (Gong et al., 2008; Trauner et al., 2019), and so it using in patients with PBC associated pruritus was limited. Systematic reviews and meta-analyses on UDCA showed that it improved the levels of serum ALP and γ-GGT, but there were no significant effects on pruritus, fatigue, or reduction in adverse events in PBC (Gong and Gluud, 2005). Similar results were obtained for methotrexate (Guo et al., 2015), fenofibrate (Zhang et al., 2015), and bezafibrate (Gong et al., 2007). Cyclosporin A may significantly improving pruritus, but it had significant side effects compared with placebo (Gerussi et al., 2021). However, we found that these studies did not consider pruritus as a primary outcome measure, and we need an effective and safe pharmacological intervention for pruritus associated with PBC was yet unmet.

Therefore, we conduct this systematic review and meta-analysis to find the effective and safety pharmacological intervention for managing pruritus in PBC.

### Pruritus (relief rate)

This meta-analysis of 23 RCTs found that UDCA, methotrexate and GSK2330672 improved pruritus (comparing the pruritus relief rate before and after treatment). OCA may increase the risk of pruritus, hence it was not recommended for patients suffering from PBC pruritus. However, due to the limited number of studies, it was suggested that more RCTs be conducted to understand their role in improving the symptom of itching (Specific data are summarized in Table 2).

### Serum alkaline phosphatase and γ-glutamyl transpeptidase

Serum ALP and γ-GGT were two important indicators for the diagnosis and prognosis of PBC^[57]^, while these also serve as important markers to diagnose the existence of cholestasis. We analyzed the effects of drug intervention on ALP and γ-GGT. All the included studies reported that UDCA, OCA, rifampicin, cyclosporine, NGM282, seladelpar and colchicine may improve blood ALP. Further, UDCA, bezafibrate, OCA, rifampicin and NGM282 may improve blood γ-GGT. We found that rifampicin can significantly reduce the blood ALP level with low heterogeneity, while UDCA and OCA have high heterogeneity in reducing the level of ALP. Although UDCA and bezafibrate reduce the level of blood γ-GGT, they have high heterogeneity. Rifampicin and NGM282 were equally efficacious in reducing blood ALP and γ- GGT, which was an area worthy of further study.

### Adverse events

The existing evidence showed that a medium dose of UDCA (13–15 *mg/kg/day*) and malotilate may have the benefit of reducing the incidence of adverse events (*p* < 0.05), and these studies showed low heterogeneity. It was also found that the other drugs that inculded did not significantly raising the incidence of adverse events compared with placebo (*p* > 0.05).

## Conclusion

In this study, we attempted to evaluate the efficacy and safety of drug interventions in the treatment of PBC-associated pruritus. It was found that UDCA, methotrexate, and GSK2330672 may improve pruritus, but due to the existence of literature quality and heterogeneity of the included drugs, we cannot recommend some therapeutic drugs in line with clinical practice. Notably, with the continuous accumulation of high-quality clinical trial evidence of some emerging drugs such as bezafibrate and ileal apical bile acid transporter inhibitors (GSK2330672), perhaps in the near future, PBC-related pruritus will get more high-quality evidence and standardized treatment, and the rate of utilization of liver transplantation will become lower.

## Data Availability

The original contributions presented in the study are included in the article/Supplementary Material, further inquiries can be directed to the corresponding author.
